# Rapid quantitation of NAD^+^/NADH and NADPH/NADP^+^ with mass spectrometry by using calibration constants

**DOI:** 10.1016/j.redox.2025.103953

**Published:** 2025-11-29

**Authors:** Qiuyuan Guo, Mike Lingjue Wang, Michaela Schwaiger-Haber, Xiangfeng Niu, Shanshan Zhang, Leah P. Shriver, Gary J. Patti

**Affiliations:** a Departments of Chemistry, Genetics, and Medicine, Washington University, St Louis, MO, USA; b Center for Mass Spectrometry and Metabolic Tracing, Washington University, St Louis, MO, USA

**Keywords:** Nicotinamide adenine dinucleotide, Nicotinamide adenine dinucleotide phosphate, NAD^+^/NADH, NADPH/NADP^*+*^, LC/MS, Calibration constants, Metabolomics

## Abstract

NAD^+^/NADH and NADPH/NADP^+^ are important indicators of cellular redox status, but they cannot be reliably calculated from the relative intensities of mass spectrometry signals alone. Establishing accurate redox ratios by mass spectrometry has historically required converting relative signal intensities into absolute concentrations, which is a time-consuming process that limits rapid analysis. Here, we describe a simpler strategy to determine NAD^+^/NADH and NADPH/NADP^+^ by using mass spectrometry. While ionization is strongly influenced by factors such as instrument drift and sample type, we discovered that the relative signal intensities of oxidized and reduced cofactors change at the same rate across experiments performed on the same mass spectrometer. That rate can be experimentally determined and expressed as a calibration constant. Using calibration constants, relative intensities of mass spectrometry signals can be rapidly transformed into accurate redox ratios without the use of authentic standards or isotopically labeled internal standards. We present a metabolomics workflow to measure NAD^+^/NADH and NADPH/NADP^+^ by using calibration constants and compare the results to other methods, including commercial colorimetric assays. Although colorimetric assays are the most widely used, we demonstrate that mass spectrometry quantitation with calibration constants provides more accurate results.

## Graphical abstract

**Figure:**
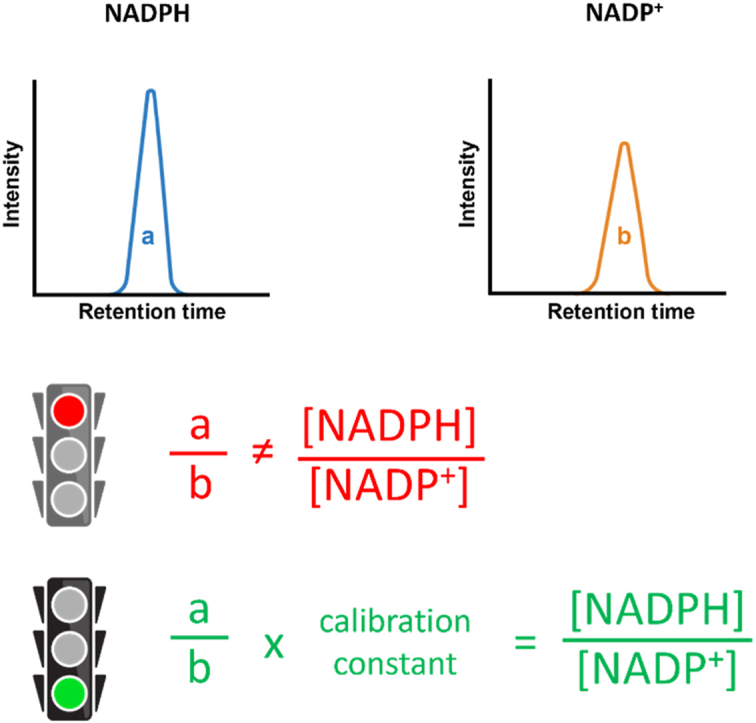

## 1 Introduction

The ratio of oxidized to reduced nicotinamide adenine dinucleotide (NAD^+^/NADH) and reduced to oxidized nicotinamide adenine dinucleotide phosphate (NADPH/NADP^+^) reflect the redox state of a biological system [1]. NAD^+^ and NADP^+^ function as electron acceptors, oxidizing target molecules via hydride transfer. NADH and NADPH, on the other hand, act as electron donors and reduce substrates [2,3]. Although NAD and NADP only differ by a phosphate group at the 2’ position of ribose, they serve distinct biochemical roles. NAD is primarily involved in energy metabolism, whereas NADP is essential for reductive biosynthesis and protection against oxidative stress [4,5]. Maintaining the ratio of NAD^+^ to NADH and NADPH to NADP^+^ requires that oxidation and reduction reactions be balanced. The rate at which NAD^+^ is reduced to NADH in glycolysis, for example, must match the rate at which NADH is oxidized by the electron transport chain or lactate dehydrogenase [6]. When oxidation of a cofactor outpaces its reduction, or vice versa, then the redox state of a biological system shifts. Alterations in NAD^+^/NADH and NADPH/NADP^+^ have been associated with a number of different pathological phenotypes including ischemia, diabetes, neurodegenerative diseases, aging, hepatic steatosis, and cardiovascular dysfunction [[7], [8], [9], [10], [11], [12], [13]]. Accordingly, assessment of each redox ratio provides important biochemical insight and is of broad interest to researchers across many fields.

To date, three experimental strategies have been primarily applied to measure redox ratios. The first uses genetically encoded probes such as SoNar to monitor redox states in living cells [[14], [15], [16]]. Genetically encoded systems provide high sensitivity and involve minimal sample preparation but they must be customized for each biological system, which is a major disadvantage limiting broad applicability. The second option is to use a colorimetric assay kit to quantify NAD^+^/NADH or NADPH/NADP^+^ [[17], [18], [19], [20]]. Here, samples are mixed with a probe that produces a colored product when it reacts with a cofactor. The approach requires that a standard curve be generated for each experiment to convert the intensity of color produced to the amount of cofactor present in the sample. Given that the dynamic range of colorimetric kits is only about one order of magnitude, the assay often needs to be performed multiple times on diluted samples to ensure that both NAD(P)^+^ and NAD(P)H can be reliably quantitated. The third strategy involves applying liquid chromatography/mass spectrometry (LC/MS) to measure oxidized and reduced cofactors directly [[21], [22], [23], [24]]. LC/MS has a wide dynamic range but, like colorimetric assays, has required a calibration curve for accurate quantitation of redox ratios. Because NAD(P)^+^ and NAD(P)H ionize with different efficiencies, equal concentrations of oxidized and reduced cofactor do not produce equal LC/MS signal intensities. Thus, relative LC/MS signal intensities must be converted into absolute concentrations by using authentic standards of each cofactor measured across a range of concentrations. The process is time consuming and impractical to perform rigorously with isotopically labeled standards due to their limited availability and cost. Moreover, it is technically complicated by the chemical instability of NAD and NADP [23]. As a result, even though oxidized and reduced cofactors are routinely measured in standard metabolomics workflows, redox ratios cannot be directly inferred from the data and LC/MS has not been widely adopted for quantitation of NAD^+^/NADH and NADPH/NADP^+^.

In this work, we set out to develop a strategy for measuring redox ratios that would take advantage of the chemical specificity and wide dynamic range of LC/MS but avoid the need to analyze authentic standards with each experiment. Our goal was to be able to extract reliable redox ratios from standard metabolomics datasets, without the burden of absolute quantitation. The approach we introduce is based on our discovery that ionization efficiencies change proportionately between oxidized and reduced cofactors across different experiments performed on the same mass spectrometer. The signal intensities of oxidized and reduced cofactors can therefore be converted into accurate redox ratios by using experimentally determined calibration constants. We describe a metabolomics workflow to measure NAD and NADP, derive their calibration constants, and then rapidly determine redox ratios without using authentic standards. For several types of biological samples, we demonstrate that our approach outperforms colorimetric assays.

## 2 Materials and methods

### 2.1 Chemicals

The following analytical standards were purchased from MilliporeSigma (St. Louis, MO): β-NADP^+^ disodium salt (97 %, 93205), β-NADPH tetrasodium salt (97 %, N7505), β-NAD hydrate (99 %, N1636), β-NADH (97 %, N8129), and Bisphenol F (51453). Water, acetonitrile (ACN), and methanol (MeOH) were purchased as LC/MS-grade solvents from Honeywell Burdick & Jackson (Muskegon, MI, USA). LC/MS-grade ammonium acetate (49638), ammonium bicarbonate (73594), and dimethyl sulfoxide (85190) were purchased from MilliporeSigma (St. Louis, MO). LC/MS-grade ammonium hydroxide (44273) and formic acid (56302) were purchased from Honeywell Burdick & Jackson (Muskegon, MI, USA). Cell-culture media and reagents were purchased from Thermo Fisher (Mountain View, CA).

### 2.2 Extraction and LC/MS analysis

Measuring the levels of NAD^+^, NADP^+^, NADH, and NADPH by LC/MS involves multiple steps. Oxidized and reduced cofactors must first be extracted from cells and tissues. The extracted samples may then be stored prior to LC/MS evaluation. A potential concern is that oxidized and reduced cofactors are interconverted or degraded during this process.

A prior report by Lu and colleagues established that NAD^+^, NADP^+^, NADH, and NADPH are most reliably extracted from cells and tissues by using 40:20:20 ACN:MeOH:H_2_O with 0.1 M formic acid [23]. The addition of 0.1 M formic acid is necessary for quenching enzymatic activity to minimize the interconversion of oxidized and reduced cofactors, but the samples must be quickly neutralized after extraction to avoid acid-catalyzed degradation of NADH and NADPH (Supplementary Fig. 1A–B). In the current study, we used this validated extraction workflow. To determine how long cofactors extracted under such conditions are stable for storage, we prepared stock solutions of each cofactor using authentic standards and diluted them by adding 2:2:1 ACN:MeOH:H_2_O (v/v/v) with 0.1 M formic acid. After 5 min, the pH of the solution was adjusted to 8.5 by adding 2 M ammonium bicarbonate. The samples were then immediately analyzed by LC/MS, stored at 4 °C for 24 h prior to LC/MS analysis, or stored at −80 °C for 14 days prior to LC/MS analysis. For a control, an identical experiment was performed without adding ammonium bicarbonate. As expected based on the findings of Lu et al., without acid neutralization, NADH and NADPH degraded quickly (Supplementary Fig. 1A–B). Acid neutralization did not prevent cofactor degradation over 14 days, but it did effectively minimize cofactor degradation for 24 h (Supplementary Fig. 1C–D). Like Lu et al., we also found that both oxidized and reduced cofactors degraded during drying (Supplementary Fig. 1E). As such, for all of the experiments performed in this study, cofactors were extracted by incubating samples for 5 min in 2:2:1 ACN:MeOH:H_2_O with 0.1 M formic acid, followed by acid neutralization with 2 M ammonium bicarbonate solution (23:2 v/v to achieve a final pH of 8.5). No drying steps were used in our workflow and all of our samples were measured by LC/MS within 24 h of extraction (Supplementary Fig. 2).

To improve quantitation, we developed a liquid chromatography method that achieved baseline separation of NAD^+^, NADP^+^, NADH, and NADPH (Supplementary Fig. 3A). With the exception of NADPH, [M − H]^-^ was the dominant ion species observed for each. NADPH formed two dominant ion species, [M − H]^-^ and [M − 2H]^2-^. For quantitation, we used the sum of the peak intensities of [M − H]^-^ and [M − 2H]^2-^ for NADPH and the peak intensity of [M − H]^-^ alone for other cofactors. The lower limit of quantitation (LLOQ) for each of the four redox cofactors with our LC/MS method was determined to be approximately 0.02 μM. The linear range for each cofactor was determined to be 0.02 to >50 μM, which is two orders of magnitude higher than colorimetric assays (Supplementary Table 1).

### 2.3 Cell culture

The human colorectal carcinoma cell line HCT116 and the near-haploid cell line HAP1 were obtained from Horizon Discovery (Cambridge, UK). The human cervical cancer cell lines SiHa, ME180, and C33A were obtained from ATCC (Manassas, VA). All cell-culture experiments were performed under standard conditions of 37 °C and 5 % CO_2_. HAP1 cells were cultured in Iscove's Modified Dulbecco's Medium (IMDM) with 20 % FBS. ME180, HCT116, and SiHa cells were cultured in Dulbecco's Modified Eagle Medium (DMEM) with 10 % FBS. C33A cells were cultured in Eagle's Minimum Essential Medium (EMEM) with 10 % FBS.

### 2.4 Metabolite extraction for cultured cells

Cells were harvested at 80–90 % confluence by removing the media and washing twice with 37 °C phosphate buffered saline (PBS). For cells cultured in 100 mm plates, 460 μL of cold extraction buffer (2:2:1 ACN:MeOH:H_2_O with 0.1 M formic acid, 4 °C) was uniformly distributed throughout the plate. Metabolism was quenched by incubation on ice for 5 min. Next, 40 μL of 2 M ammonium bicarbonate was added to the extraction buffer to adjust the pH to 8.5. The cells were then scraped from the plate and transferred to a 1.5 mL Eppendorf tube. Three cycles of freezing in liquid nitrogen (1 min), thawing in a 25 °C water bath (10 s), sonication (10 min), and vortexing (30 s) were performed for metabolite extraction, followed by incubation at −20 °C for 1 h. The extract was then centrifuged at 14,000 g at 4 °C for 10 min. The supernatant was transferred to LC/MS vials for same-day analysis.

### 2.5 Quantitative analysis of redox ratios by LC/MS

Triple quadrupole (QQQ) and quadrupole time-of-flight (QTOF) mass spectrometers were used in this work. The QQQ (6460, Agilent, Santa Clara, CA) and QTOFs (6530, 6540, and 6545, Agilent, Santa Clara, CA) were coupled to an Agilent 1290 UHPLC system (Agilent, Santa Clara, CA). Metabolite separation was achieved with a hydrophilic interaction liquid chromatography (HILIC) method by using a charge modulated diol polymeric column (100 mm × 2.1 mm, 5 μm, 200 Å, iHILIC-(P) Classic, HILICON, Sweden). Mobile phase A consisted of 95 % water and 5 % ACN with 10 mM ammonium acetate and 10 mM ammonium hydroxide. Mobile phase B consisted of 95 % ACN and 5 % water. The separation of 3 μL of sample was performed at a flow rate of 200 μL/min with the following linear gradient: 0 min, 95 % B; 1 min, 95 % B; 5 min, 30 % B; 15 min, 0 % B; 20 min, 0 % B; 25 min, 95 % B; 30 min, 95 % B. With this LC gradient, we achieved baseline separation of all four cofactors (Supplementary Fig. 3A).

MS analysis was performed in negative mode by using electrospray ionization. Identifications were supported by accurate mass as well as MS/MS data and retention times from authentic standards. QQQ experiments were only used for the analysis of standard mixtures to demonstrate the stability of calibration constants over time on a different instrument type. Selected ion monitoring (SIM) mode was used for quantitation and MRM mode was used for identification. While multiple reaction monitoring (MRM) mode is preferred for quantitation when evaluating complex biological matrices, SIM mode enables more efficient ion transmission in cases where pure standards are used and selectivity is not needed [25]. To ensure that SIM quantitation was not affected by interfering ions, we validated the purity of each standard with high-resolution mass spectrometry and confirmed that our solvents did not produce overlapping background signals. All biological samples were run on QTOF instruments. As noted above, [M − H]^-^ ions were used to quantitate NAD^+^ (*m/z* = 662.1018), NADH (*m/z* = 664.1175), and NADP^+^ (*m/z* = 742.0682). For NADPH, [M − H]^-^ (*m/z* = 744.0838) and [M − 2H]^2-^ (*m/z* = 371.5383) were summed for quantitation. Throughout this work, we denote the detected negative-mode ions of NAD^*+*^ and NADP ^*+*^ as [M − H] ^-^, where the molecular ion M is defined as the neutral molecule (Supplementary Fig. 3B). M can also be defined as the +1 charged molecule (Supplementary Fig. 3C), in which case the corresponding negative-mode ion is [M − 2H] ^-^. Of note, the calculated *m/z* is identical with both notations. The notation used does not affect data processing.

All experiments used Agilent JetStream electrospray ionization with the following source conditions: dry gas: 10 L/min, sheath gas temperature: 300 °C, sheath gas flow: 12 L/min, nozzle voltage: 1000 V, nebulizer: 42 psi, capillary voltage: 3500 V. For QQQ experiments, an optimal fragmentor voltage was set for each analyte: NAD^+^, 110 V; NADH, 190 V; NADP^+^, 100 V; NADPH-[M − H]^-^, 200 V; NADPH-[M − 2H]^2-^, 80 V. The [M − H]^-^ precursor ion was measured by SIM. Two fragment ions were measured for each analyte by MRM as qualifier ions: NAD^+^, *m/z* 662→540 at CE 10 V and *m/z* 662→273 at CE 40 V; NADH, *m/z* 664→408 at CE 30 V and *m/z* 664→79 at CE 40 V; NADP^+^, *m/z* 742→620 at CE 10 V and *m/z* 742→408 at CE 30 V; and NADPH, *m/z* 371.5→304 at CE 10 V, 371.5→134 at CE 20 V. For QTOF experiments, MS full scan was used with a scan rate of 1 spectrum/s and a mass range of *m/z* 80–1000. Given that [M − H]^-^ was the dominant ion species observed for NAD^+^ (*m/z* = 662.1018), NADH (*m/z* = 664.1175), and NADP^+^ (*m/z* = 742.0682), [M − H]^-^ ions were used as a quantifier. For NADPH, [M − H]^-^ (*m/z* = 744.0838) and [M − 2H]^2-^ (*m/z* = 371.5383) were summed for quantitation, because NADPH formed two dominant ion species [M − H]^-^ and [M − 2H]^2-^. LLOQ was defined as the lowest concentration of an analyte producing a peak with a signal-to-noise ratio (S/N) ≥10. At 0.02 μM, the measured S/N values were as follows: NAD^+^ = 10.8, NADH = 14.4, NADP^+^ = 10.7, and NADPH = 11.5, as determined by the Agilent MassHunter Qualitative Analysis software.

### 2.6 External calibration curves

Authentic standards of NAD^+^, NADH, NADP^+^, and NADPH were dissolved in 2:2:1 acetonitrile:methanol:water with 0.1 M formic acid and mixed with 2 M ammonium bicarbonate aqueous solution (v/v = 23:2) to prepare a 400 μM stock solution. The stock solution was serially diluted by using the same solvent mixture to yield calibrant solutions at 0.5, 1, 2, 5, and 10 μM. Each were analyzed by using the LC/MS method described above. A standard curve was generated by plotting peak area against concentration, and the slope, corresponding to k_NAD(P)_, was determined by using linear regression.

### 2.7 Standard-addition method

Using the protocol described above, extracts were generated from HCT116 cells, HAP1 cells, HeLa cells, SiHa cells, and zebrafish liver. Authentic NAD^+^, NADP^+^, NADH, and NADPH standards were dissolved in 2:2:1 acetonitrile:methanol:water with 0.1 M formic acid and mixed with 2 M ammonium bicarbonate aqueous solution (v/v = 23:2) to prepare a stock solution of 400 μM. The stock solution was further diluted to achieve 5, 10, 20, and 50 μM standard mixture solutions. Then 5 μL of each standard mixture or blank (i.e., extraction solvent without cofactor) was spiked into 45 μL of extract from HCT116 cells, HAP1 cells, HeLa cells, SiHa cells, or zebrafish liver. Samples were transferred to LC/MS vials for same-day analysis.

### 2.8 Unlabeled nucleotides spiked in uniformly labeled yeast

Uniformly ^13^C-labeled (99 %) metabolite yeast extract (ISO1, Cambridge Isotope Laboratories) was reconstituted in 2 mL LC/MS-grade water and further diluted 1:10 in neutralized extraction solvent. ISO1 yeast provides a biological extract where all metabolites are uniformly–^13^C labeled. This means that it is without endogenous, unlabeled cofactors. Different amounts of unlabeled NADP^+^ and NADPH standards were dissolved in neutralized extraction solvent to prepare standard mixtures with different ratios of unlabeled reduced to unlabeled oxidized cofactors (NADPH/NADP^+^ was at 1:1, 10:1, and 1:10). Next, 5 μL of each standard mixture was spiked into 45 μL yeast extract for same-day LC/MS analysis.

### 2.9 Quantitative analysis of NADPH/NADP^+^ by using colorimetric assays

Colorimetric assay kits were obtained from Abcam (ab65349) and MilliporeSigma (MAK479) for quantitative analysis of NADPH/NADP^+^ as a comparison to LC/MS-based analysis. The assays were performed following the manufacturer's protocol by using freshly reconstituted reagents. Ratios were calculated from the absorbance values by using a standard curve.

### 2.10 Cofactors spiked into HCT116 extract

Metabolites were extracted from cultured HCT116 cells as described above. An equivalent of 500 pmol of NADPH standard and 500 pmol of NADP^+^ standard was spiked into the HCT116 extract. The extract to which NADPH was spiked is referred to the as the “reduced” sample and the extract to which NADP^+^ was spiked is referred to as the “oxidized” sample. Unspiked HCT116 extract is referred to as the “control” sample. The reduced, oxidized, and control samples were analyzed by using LC/MS and colorimetric assay kits (ab65349, Abcam, Waltham, MA). The recovery % of spiked NADP^+^ and NADPH was measured by LC/MS and colorimetric assays (Supplementary Table 2).

### 2.11 Zebrafish husbandry

The Washington University Institutional Animal Care and Use Committee approved all experimental procedures (protocol 24–0312). Experiments used inbred AB strain (SjA) zebrafish, reared according to standard procedures. Zebrafish were maintained in an indoor environment with a 14:10 h light: dark circadian cycle. The water temperature, pH, and conductivity were kept at 28 ± 1 °C, 7.4 ± 0.2, and 850 ± 50 μS/cm, respectively.

### 2.12 Bisphenol F exposure

Stock solutions of bisphenol F (BPF, 10 mg/L, 100 mg/L, and 1000 mg/L) were prepared in DMSO and stored in the dark at 4 °C. Experiments were performed by using adult zebrafish (10-month old). Eight female and eight male fish were exposed to 1 μg/L, 10 μg/L, and 100 μg/L BPF or a blank control (DMSO 0.01 % v/v) in 5 L tanks for 7 weeks. During the experiment, all tanks were maintained under the same pre-experimental conditions as described above, with fish allowed to swim freely. Fish were fed with Gemma micro 300 zebrafish food (Skretting) daily at noon (4 mg/per fish) and the water in all tanks was changed every other day. After 7 weeks of exposure, fish were euthanized by using gradual cooling. Livers from zebrafish were harvested in pre-weighed 1.5 mL Eppendorf tubes, snap frozen in liquid nitrogen, and stored in −80 °C until metabolite extraction.

### 2.13 Metabolite extraction from zebrafish liver for LC/MS analysis

Frozen zebrafish liver was homogenized in pre-weighed 1.5 mL Eppendorf tubes with a disposable pestle prechilled in liquid nitrogen. After tissue disruption, tubes containing zebrafish liver were weighed to calculate the net liver weight. Cold extraction buffer (2:2:1 ACN:MeOH:H_2_O with 0.1 M formic acid, 4 °C) was added to the ground liver. For every 1 mg of liver wet weight, 40 μL of extraction solvent was added. The extraction mixture was then vortexed for 30 s and incubated on ice for 5 min. Next, 2 M ammonium bicarbonate was added to the extraction buffer to adjust the pH to 8.5. The mixture was subjected to three cycles of freezing in liquid nitrogen (1 min), thawing in a 25 °C water bath (10 s), sonication (5 min), and vortexing (30 s), followed by incubation at −20 °C for 1 h. After protein precipitation, liver extracts were centrifuged at 14,000 g at 4°C for 15 min. The supernatant was transferred to LC/MS vials for same-day analysis.

### 2.14 LC/MS-based untargeted metabolomics

Two quality-control (QC) samples were made by mixing female and male livers. A Vanquish UHPLC system was coupled to an Orbitrap ID-X Tribrid mass spectrometer (Thermo Fisher Scientific) via electrospray ionization with the following source conditions: sheath gas flow 50 arbitrary units (Arb), auxiliary gas flow 10 Arb, sweep gas flow 1 Arb, ion transfer tube temperature 300 °C, and vaporizer temperature 200 °C. The RF lens value was 60 %. Data were acquired in negative polarity with a spray voltage of 2.8 kV. MS1 data were acquired from 67 to 900 *m/z* at a resolution of 120,000 with an automatic gain control (AGC) target of 2e5 and a maximum injection time of 200 ms. MS/MS data for metabolite identification were acquired at a resolution of 15,000 with an AGC target of 2.5e4 and a maximum injection time of 70 ms.

### 2.15 Data analysis and visualization

LC/MS data were processed and analyzed by Skyline (University of Washington) [26,27]. Prism 10 (Graphpad) was used for statistical analysis and data visualization. Data are presented as mean ± SD, except for animal experiments that are shown as mean ± SEM. The statistical comparisons used for each experiment and the thresholds for statistical significance are indicated in the respective figure legends. For untargeted metabolomics data processing, peaks were annotated with Compound Discoverer 3.3 by matching MS/MS spectra of pooled QC samples to mzCloud.

## 3 Results

### 3.1 Calibration constants to quantitate redox ratios

Historically, to assess NAD^+^/NADH accurately by LC/MS, the absolute concentrations of oxidized and reduced cofactors have been determined by using the following equations:

Equation 1 $$\left[{\text{NAD}}^{+}\right]={\text{Response}}_{{\text{NAD}}^{+}}/k_{{\text{NAD}}^{+}}$$

Equation 2 $$\left[\text{NADH}\right]={\text{Response}}_{\text{NADH}}/k_{\text{NADH}}$$

Here, [NAD^+^] and [NADH] denote the absolute concentrations of NAD^+^ and NADH, respectively. Response_NAD+_ and Response_NADH_ are the total integrated areas of the LC/MS peaks for NAD^+^ and NADH, respectively. Finally, k_NAD+_ and k_NADH_ are the response coefficients derived from the slope of standard calibration curves. The equations assume a y-intercept of zero. To create a standard calibration curve, Response_NAD+_ is plotted against [NAD^+^] and Response_NADH_ against [NADH]. As we discuss further below, it is best to generate calibration curves within biological matrices. Initially, however, peak areas from different concentrations of isolated standards were measured and response coefficients determined based on the slope of the data. The same equations can be written for NADPH and NADP^+^.

Equation 3 $$\left[\text{NADPH}\right]={\text{Response}}_{\text{NADPH}}/k_{\text{NADPH}}$$

Equation 4 $$\left[{\text{NADP}}^{+}\right]={\text{Response}}_{{\text{NADP}}^{+}}/k_{{\text{NADP}}^{+}}$$

The equations can be rearranged to solve for NAD^+^/NADH and NADPH/NADP^+^ as follows:

Equation 5 $$\frac{\left[{\text{NAD}}^{+}\right]}{\left[\text{NADH}\right]}=\frac{{\text{Response}}_{{\text{NAD}}^{+}}/k_{{\text{NAD}}^{+}}}{{\text{Response}}_{\text{NADH}}/k_{\text{NADH}}}$$

Equation 6 $$\frac{\left[\text{NADPH}\right]}{\left[{\text{NADP}}^{+}\right]}=\frac{{\text{Response}}_{\text{NADPH}}/k_{\text{NADPH}}}{{\text{Response}}_{{\text{NADP}}^{+}}/k_{{\text{NADP}}^{+}}}$$

The expression can then be re-written as:

Equation 7 $$\frac{\left[{\text{NAD}}^{+}\right]}{\left[\text{NADH}\right]}=\frac{{\text{Response}}_{{\text{NAD}}^{+}}}{{\text{Response}}_{\text{NADH}}}\times \frac{k_{\text{NADH}}}{k_{{\text{NAD}}^{+}}}$$

Equation 8 $$\frac{\left[\text{NADPH}\right]}{\left[{\text{NADP}}^{+}\right]}=\frac{{\text{Response}}_{\text{NADPH}}}{{\text{Response}}_{{\text{NADP}}^{+}}}\times \frac{k_{{\text{NADP}}^{+}}}{k_{\text{NADPH}}}$$

We speculated that the ratio of the response coefficients (k_NADH_/k_NAD+_ and k_NADP+_/k_NADPH)_ would be conserved among experiments performed on the same mass spectrometer. We define each ratio as a calibration constant, C.

Equation 9 $$C_{\text{NAD}}=\frac{k_{\text{NADH}}}{k_{{\text{NAD}}^{+}}}$$

Equation 10 $$C_{\text{NADP}}=\frac{k_{{\text{NADP}}^{+}}}{k_{\text{NADPH}}}$$

**Fig. 1:**
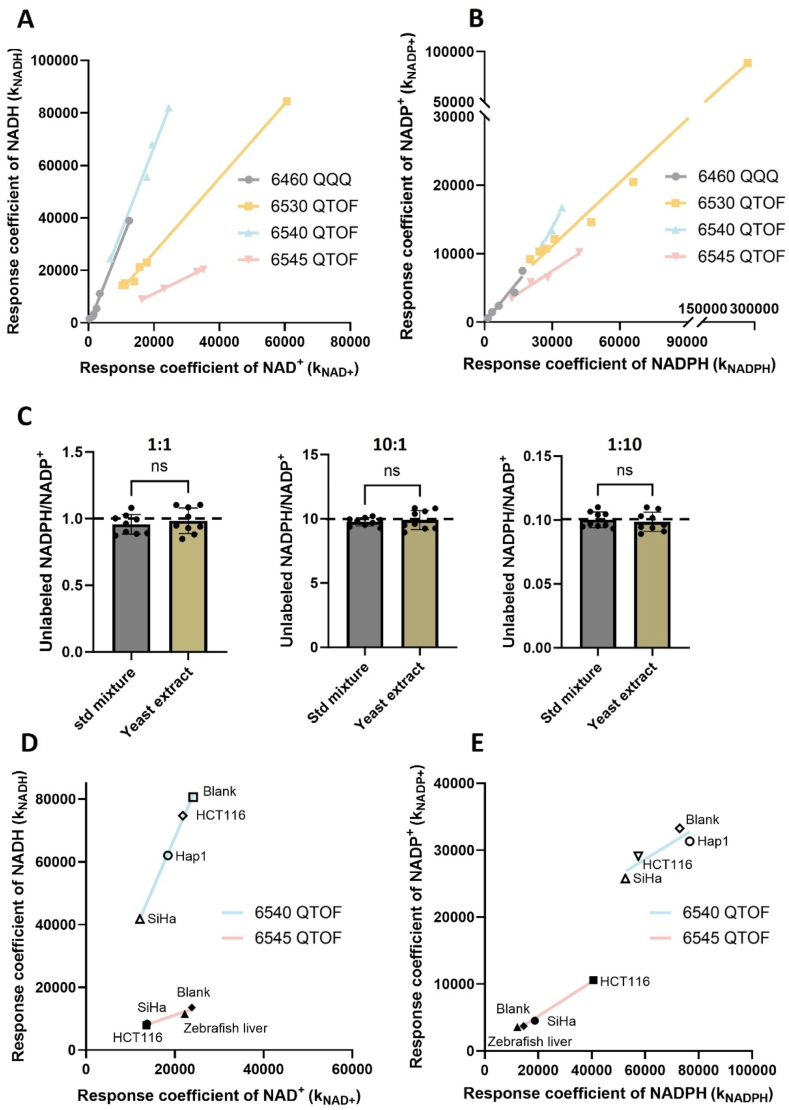
**Calibration constants remain the same in spite of instrument drift and matrix effects.** (A) Response coefficients k_NADH_ vs. k_NAD+_ and **(B)** k_NADP +_ vs. k_NADPH_ as measured by four different mass spectrometers from mixtures of authentic standards. **(C)** Unlabeled NADPH/NADP^+^ as measured from mixtures of authentic standards (standard mixtures) or uniformly ^13^C-labeled yeast extracts (yeast extract). Data were acquired on an Agilent 6545 QTOF, n = 9. **(D)** Response coefficients k_NADH_ vs. k_NAD+_ and **(E)** k_NADP +_ vs. k_NADPH_ as measured from different matrices on an Agilent 6540 QTOF and 6545 QTOF. All data are presented as means ± SD. ns = not significant by unpaired two-tailed t tests.

To validate that C_NAD_ and C_NADP_ are indeed constants on a particular mass spectrometer, we analyzed isolated standards of NAD^+^, NADP^+^, NADH, and NADPH at different concentrations on four mass spectrometers. The experiments were performed over three years to assess instrument drift, during which time data were collected with the same method. Response coefficients for each oxidized and reduced cofactor were determined from the slopes of standard curves. We found that although the response coefficients changed over time, a highly linear relationship persisted between k_NADH_ and k_NAD+_ (Fig. 1A). A similarly high correlation was observed between k_NADP+_ and k_NADPH_ (Fig. 1B). From the data, we calculated the calibration constants (Supplementary Table 3). Irrespective of the time at which the data were collected, C_NAD_ and C_NADP_ remained constant with a low Relative Standard Deviation (RSD%). The results show that the signal intensities of oxidized and reduced cofactors vary with instrument drift, but they do so proportionately, allowing correction by using calibration constants.

### 3.2 Calibration constants are conserved across different biological matrices

In LC/MS, the efficiency at which an isolated chemical standard ionizes may not match the efficiency at which the same chemical ionizes when measured from a complex biological specimen. The presence of other molecules in a sample can either suppress or enhance ionization of the target chemical, thereby altering its signal intensity independent of a change in its concentration. The influence that a sample matrix has on a target chemical's ionization and detection, often referred to as “matrix effects”, poses a major challenge for quantitation [[28], [29], [30]]. We aimed to evaluate whether matrix effects influence oxidized and reduced cofactors similarly, meaning that calibration constants could be used to transform peak areas from raw LC/MS data into accurate redox ratios.

One way to assess the impact of matrix effects is by spiking isotopically labeled standards into a biological sample [31,32]. Labeled and unlabeled chemicals ionize with the same efficiency, but the differences in their mass enable them to be differentiated by LC/MS. Assuming that there is no labeled chemical in the original sample, the signal intensity of a target can be quantitatively assessed over a wide concentration range by spiking isotopic standard into the biological matrix.

The availability of isotopically labeled NAD and NADP standards is limited. Thus, it was not readily feasible to assess the relationship between signal intensity and concentration by spiking isotopically labeled standards into an unlabeled biological sample in this study. As an alternative, we spiked unlabeled NADPH and NADP ^*+*^ standards at a ratio of 1:1, 10:1, or 1:10 into a metabolite extract obtained from uniformly ^13^C-labeled yeast. As a control, unlabeled NADPH and NADP ^*+*^ standards were spiked into blanks (i.e., samples without any biological material) at the same ratios in parallel. Given that the yeast extract was enriched with ^13^C, this experimental design enabled us to distinguish spiked NADP^+^ and NADPH (unlabeled) from endogenous NADP^*+*^ and NADPH (labeled) by using mass spectrometry. After analyzing the samples on an Agilent 6545 QTOF, we used Equation (8) to calculate unlabeled NADPH/NADP^*+*^ in yeast extract and blanks (Fig. 1C). Peak areas were obtained from the QTOF data and the calibration constant was taken from Supplementary Table 3. The ratios obtained from yeast and blanks were not statistically different, indicating that calibration constants are minimally affected by biological matrix. We also calculated the relative error between unlabeled NADPH/NADP ^*+*^ as measured by LC/MS and the true ratios based on the spike-in values. In all cases, the relative error was less than 5 % (Fig. 1C and Supplementary Table 4). Taken together, these data demonstrate the reliability of our method and indicate that C_NAD_ and C_NADP_ remain constant across different matrices.

To obtain further support that calibration constants are minimally influenced by matrix effects, we aimed to assess C_NAD_ and C_NADP_ from multiple different sample types ranging from mammalian cells in culture to tissue. Given the limited availability of isotopically labeled cofactor standards and the challenges of uniformly ^13^C-labeling mammalian samples, we used the standard-addition method to infer response coefficients [33]. In brief, increasing concentrations of unlabeled cofactors were spiked into metabolite extracts obtained from HCT116 cells, HAP1 cells, SiHa cells, or liver tissue. Using data from two mass spectrometers (Supplementary Fig. 4 and Supplementary Fig. 5), the concentration of cofactor spiked into the sample was plotted versus peak area. Response coefficients for NAD^+^, NADP^+^, NADH, and NADPH were then determined from the slope of the regression line. Notably, the response coefficients for each pair of oxidized and reduced cofactors changed proportionately between datasets collected from the same instrument (Fig. 1D and E). The results demonstrate that C_NAD_ and C_NADP_ are constant across all of the samples measured on a given mass spectrometer (Supplementary Table 5 and Supplementary Table 6).

### 3.3 Quantitative comparison of LC/MS methods

There are three LC/MS-based approaches that can be applied to assess NAD^+^/NADH and NADPH/NADP^+^. The first is to create a ratio directly from the peak areas of NAD^+^, NADP^+^, NADH, and NADPH as quantitated from the raw LC/MS data [24]. The second is to convert the peak areas of each oxidized and reduced cofactor to an absolute concentration, and then use those values to create a ratio [34]. The third is to use the strategy we introduce here that converts raw data to ratios by using calibration constants, without determining absolute concentrations. We sought to compare the performance of each.

**Fig. 2:**
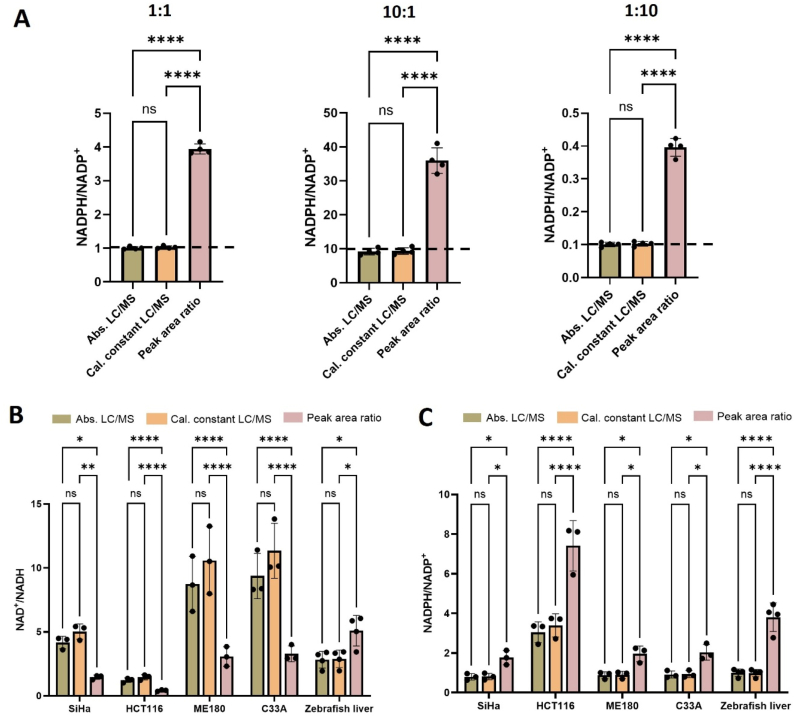
**Redox ratios determined by calibration constants are just as accurate as those determined by absolute quantitation.(A)** Authentic standards of NADPH and NADP^+^ were used to create samples with 1:1, 10:1, and 1:10 ratios. NADPH/NADP^+^ was determined by using absolute concentrations of oxidized and reduced cofactors (Abs. LC/MS), calibration constants (Cal. Constant LC/MS), or the peak areas from the raw data directly (Peak area ratio). The data were acquired on an Agilent 6545 QTOF, n = 4. To obtain absolute concentrations, standard curves were created. The calibration constant was obtained from Supplementary Table 2. **(B)** NAD^+^/NADH as measured from four cancer cell lines on an Agilent 6540 QTOF (n = 3). Zebrafish liver tissue was measured on an Agilent 6545 QTOF (n = 4). **(C)** NADPH/NADP^+^ as measured from four cancer cell lines on an Agilent 6540 QTOF, n = 3. Zebrafish liver tissue was measured on an Agilent 6545 QTOF, n = 4. All data are presented as means ± SD. ns = not significant, ∗*p* < 0.05, ∗∗*p* < 0.01, ∗∗∗∗*p* < 0.0001 by one-way ANOVA followed by Dunnett's T3 multiple comparison tests (A) or two-way ANOVA followed by Sidak's multiple comparison tests (B,C).

To establish a ground truth, we mixed authentic standards of NADPH and NADP^+^ at a ratio of 1:1, 10:1 and 1:10. We then measured the solutions by using each of the three LC/MS-based approaches outlined above (Fig. 2A). As expected, redox ratios determined directly from peak areas of oxidized and reduced cofactors were not accurate. NADPH/NADP^+^ was over-estimated by nearly 4x in each of the samples, demonstrating that differences in ionization efficiency prevent direct comparison of relative peak areas (Supplementary Table 7). In contrast, assessment of NADPH/NADP^+^ from the absolute concentrations of each oxidized and reduced cofactor yielded redox ratios with an average relative error less than 5 %. Absolute quantitation by LC/MS is a well-established and reliable method, so the accuracy of these results was anticipated. Of note, however, calibration constants gave equally accurate results.

To extend the analysis to biological samples, we evaluated four different cancer cell lines as well as liver tissue from zebrafish. We used the standard-addition method to quantitate the absolute concentrations of NAD^+^, NADP^+^, NADH, and NADPH from each sample. The redox ratios we obtained by using these absolute concentrations were not statistically different than the redox ratios that we obtained by using raw peak areas normalized with calibration constants (Fig. 2B and C). Using the raw peak areas directly, however, yielded redox ratios with relative errors as high as 250 %. These results support that calibration constants can be used to determine redox ratios with accuracy that approximates absolute quantitation methods.

### 3.4 Quantitative comparison of LC/MS to colorimetric assays

**Fig. 3:**
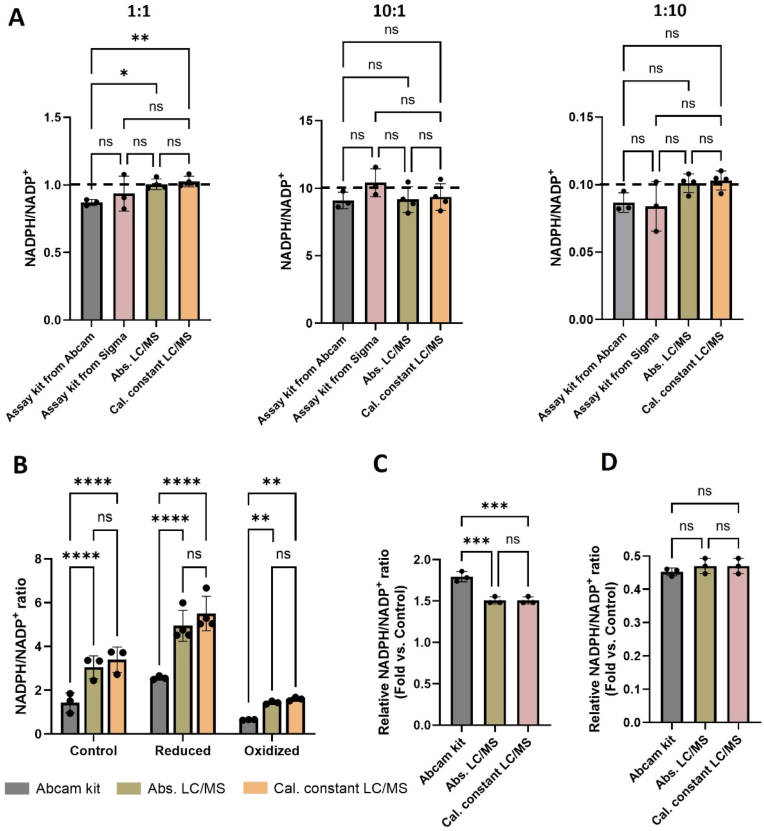
**Measurements of cellular NADPH/NADP**^**+**^**are different between colorimetric kits and LC/MS. (A)** Authentic standards of NADPH and NADP^+^ were used to create samples with 1:1, 10:1, and 1:10 ratios. Each sample was analyzed by two colorimetric kits (assay kit from Abcam and assay kit from MilliporeSigma) as well as LC/MS. For LC/MS, redox ratios were determined from absolute concentrations (Abs. LC/MS) or by using calibration constants (Cal. constant LC/MS). LC/MS data were acquired from an Agilent 6545 QTOF, n = 3–4. **(B)** NADPH/NADP^+^ as measured from HCT116 cells by using colorimetric kits or by using LC/MS methods. The LC/MS data were acquired from an Agilent 6540 QTOF, n = 3–4. To create a “reduced” sample, 500 pmol of NADPH standard was spiked into the HCT116 metabolite extract. To create an “oxidized” sample, 500 pmol of NADP^+^ was spiked into the HCT116 metabolite extract. Absolute concentrations of NADPH and NADP^+^ were determined by using external calibration curves. **(C)** Relative fold changes in NADPH/NADP^+^ between control and reduced samples, n = 3. Colorimetric data were based on measurements made by using the Abcam kit. **(D)** Relative fold changes in NADPH/NADP^+^ between control and oxidized samples, n = 3. All data are presented as means ± SD. ns = not significant, ∗*p* < 0.05, ∗∗*p* < 0.01, ∗∗∗*p* < 0.001, ∗∗∗∗*p* < 0.0001 by two-way ANOVA followed by Turkey's multiple comparison tests (B) or one-way ANOVA followed by Turkey's multiple comparison tests (A, C, D).

Redox ratios are most commonly assessed by using colorimetric assay kits. To date, the accuracy of colorimetric kits has not been quantitatively compared to LC/MS. We evaluated colorimetric kits from Abcam and MilliporeSigma for measuring NADPH/NADP^+^. We first made standard mixtures of NADPH and NADP^+^ at a ratio of 1:1, 10:1, and 1:10. The samples were evaluated by each colorimetric kit and by LC/MS, with ratios being determined by either absolute concentrations or calibration constants. Overall, the results from each measurement were generally consistent and indicate similar performance for standard mixtures (Fig. 3A).

We next aimed to compare colorimetric and LC/MS measurements made on complex biological samples, which contain other molecules that could potentially influence the accuracy and reliability of quantitation. We used metabolites extracted from HCT116 cells as a control, and either spiked in 500 pmol of NADPH to create an artificially “reduced” sample or 500 pmol of NADP^+^ to create an artificially “oxidized” sample. We expected the reduced sample to have a higher NADPH/NADP^+^ value than the control and the oxidized sample to have a lower NADPH/NADP^+^ value than the control. The samples were analyzed in parallel by using the Abcam colorimetric kit following the manufacturer's protocol or by using LC/MS. For LC/MS, the ratios were determined by absolute concentrations or by using raw peak areas normalized by calibration constants. All of the measurements aligned with our expected results, with increased ratios observed in the reduced samples and decreased ratios observed in the oxidized samples (Fig. 3B). While the values measured from both LC/MS approaches were not statistically different, the colorimetric assay reported lower ratios for all sample groups. Often, alterations in redox ratios are represented as relative fold changes between conditions (i.e., NADPH/NADP+ is two-fold higher in a disease state relative to a healthy state) [35]. Fixed bias, which occurs when one method gives values that are higher or lower than those of another by a constant amount, would result in consistent fold changes between sample groups whether colorimetric or LC/MS assays were used. That is not what was observed in the analyses performed here. The data indicate that the bias between the techniques is not fixed. The relative fold change calculated between the control and reduced samples from the colorimetric data was higher compared to the relative fold change calculated from LC/MS data (Fig. 3C and D).

**Fig. 4:**
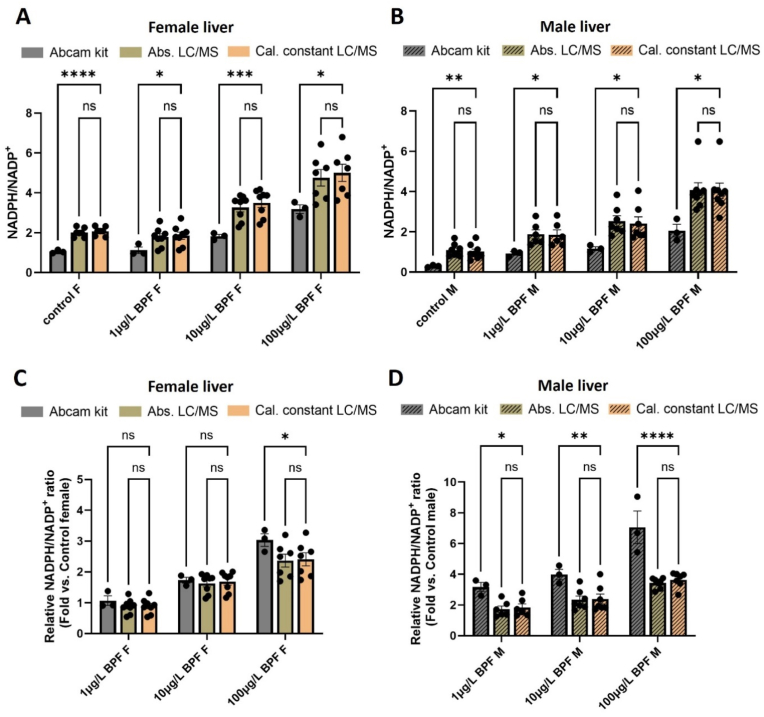
**Measurements of tissue NADPH/NADP**^**+**^**are different between colorimetric kits and LC/MS.(A)** NADPH/NADP^+^ as measured from female liver tissue by using an Abcam colorimetric kit (Abcam kit) or LC/MS. For LC/MS, redox ratios were determined from absolute concentrations (Abs. LC/MS) or by using calibration constants (Cal. constant LC/MS). Absolute concentrations were obtained by using external calibration curves or by using the standard-addition method (Supplementary Fig. 6). LC/MS data were acquired from an Agilent 6545 QTOF, n = 3–8 per condition. Female fish were treated with no BPF (control F) or increasing doses of BPF (e.g., 1 μg/L BPF F etc.). **(B)** An identical experiment was repeated with male fish, n = 3–8 per condition. **(C)** Relative fold changes in NADPH/NADP^+^ between controls and female fish treated with the indicated dosage of BPF, n = 3–8 per condition. **(D)** Relative fold changes in NADPH/NADP^+^ between controls and male fish treated with the indicated dosage of BPF, n = 3–8 per condition. All data are presented as means ± SEM. ns = not significant, ∗*p* < 0.05, ∗∗*p* < 0.01, ∗∗∗*p* < 0.001, ∗∗∗∗*p* < 0.0001 by two-way ANOVA followed by Turkey's multiple comparison tests.

To extend our analysis to tissues, we treated adult zebrafish with bisphenol F (BPF) and then evaluated NADPH/NADP^+^ from liver tissue by using Abcam's colorimetric kit and LC/MS. Fish were transferred to tanks containing BPF at 1 μg/L, 10 μg/L, or 100 μg/L for seven weeks and compared to fish that were treated with vehicle control over the same time period. Prior work using mouse models demonstrated that treatment with high levels of BPF leads to hepatic steatosis and fibrosis, which has been associated with alterations in liver NADPH/NADP^+^ [12,[36], [37], [38], [39]]. Our results from both colorimetric kits and LC/MS demonstrate that BPF exposure also alters NADPH/NADP^+^ in the liver of zebrafish (Fig. 4A and B). Notably, however, LC/MS analyses provided NADPH/NADP^+^ values that were approximately two times higher than colorimetric kits for both females and males. It is important to point out that the LC/MS results were not significantly different whether ratios were determined by absolute concentrations or by using calibration constants (Supplementary Fig. 6). As above, we similarly evaluated relative fold changes in the ratio of NADPH to NADP^+^ between the control and treatment conditions. The results provided further evidence that the difference between colorimetric and LC/MS measurements is not fixed. In all of the livers from male animals, and some of the livers from female animals, we found that colorimetric kits provided larger fold changes than LC/MS analysis (Fig. 4C and D). Finally, we should mention that an advantage of LC/MS analysis over colorimetric assays is that it provides information on a large number of metabolites beyond NAD^+^, NADP^+^, NADH, and NADPH (Supplementary Fig. 7).

## 4 Discussion

Accurate evaluation of NAD^+^/NADH and NADPH/NADP^+^ is key to understanding redox homeostasis. One strategy to assess these ratios is by measuring the abundance of oxidized and reduced cofactors by using LC/MS. A complication is that the readout of LC/MS is relative signal intensity, which is not equivalent to concentration. Due to differences in ionization efficiency, two chemicals present at the same concentration will not necessarily produce equal LC/MS peak areas. As a result, peak areas from raw data cannot be used directly to calculate NAD^+^/NADH and NADPH/NADP^+^. Although such an approach has been used in the past, we demonstrate here that it is highly unreliable and should be avoided. In some cases, raw peak areas yielded ratios with nearly 300 % error.

A more robust approach for calculating redox ratios by LC/MS is to convert relative peak areas into absolute concentrations by using the standard-addition method. The process requires analyzing biological samples in which oxidized and reduced cofactor standards have been spiked in at multiple concentrations. The experiments are tedious, time consuming, exhaust ample amounts of valuable sample material, and have to be repeated on a regular basis as laboratory conditions drift, LC/MS instrumentation is changed, or new samples are evaluated. These barriers have limited the broad applicability of the approach.

In this study, we report the discovery that the ionization efficiencies of oxidized and reduced cofactors change proportionately over time and across sample types so long as experiments are performed on the same mass spectrometer with the same method. The implication is that NAD(P) ratios based on raw peak areas and NAD(P) ratios based on absolute concentrations are related by a simple multiplication factor, which we define as the calibration constant (C_NAD_ or C_NADP_). Once a calibration constant is determined, then raw peak areas from oxidized and reduced cofactors can be rapidly converted into accurate redox ratios without having to assess absolute concentrations in each experiment. This enables reliable determination of NAD^+^/NADH and NADPH/NADP^+^ from the output of a standard untargeted metabolomics experiment, assuming an appropriate extraction method is used to prevent the interconversion or degradation of oxidized and reduced cofactors. We calculated NAD^+^/NADH and NADPH/NADP^+^ values by using both absolute concentrations and calibration constants for a variety of samples ranging from cells to tissues. In all cases, there was no significant difference between the approaches. Using authentic standards in which oxidized and reduced cofactors were mixed at defined ratios, both approaches returned values with less than 5 % error. Taken together, these findings support the conclusion that calibration constants provide a reliable strategy to assess NAD(P) redox ratios.

At least in part due to the aforementioned limitations of LC/MS, colorimetric assays have been the most widely used approach to determine NAD^+^/NADH and NADPH/NADP^+^. Our study provides a direct comparison of colorimetric assays and LC/MS. We found the quantitative performance of the two techniques to be comparable when analyzing authentic standards, but that was not the case with biological samples. For cells and tissues, we consistently observed lower NADPH/NADP^+^ values from colorimetric kits than from LC/MS data. The disparity in values did not exhibit fixed bias, meaning that fold changes (i.e., the difference in redox ratios between two sample groups) were inconsistent for colorimetric assays and LC/MS data. It is interesting to consider where the discrepancy between the techniques arises. Prior work has shown that the addition of 0.1 M formic acid during metabolite extraction is necessary for quenching enzymatic activity to minimize the interconversion of oxidized and reduced cofactors [23]. The protocols provided with colorimetric kits do not use formic acid during sample preparation and this is likely at least one source of variation between the experimental strategies. It would also explain why the results from authentic standards, which do not require quenching of enzymatic activity, are similar.

Looking ahead, it is intriguing to consider the application of calibration constants to other biologically relevant ratios that involve structurally similar metabolites such as the adenylate energy charge, reduced and oxidized glutathione, or creatine and phosphocreatine. The use of calibration constants could potentially eliminate the need for calibration curves when assessing these metabolite relationships, which would increase the information content of LC/MS-based metabolomics data without additional experimentation. Further investigation is needed to test the effectiveness of calibration constants beyond NAD^+^/NADH and NADPH/NADP^+^.

## CRediT authorship contribution statement

**Qiuyuan Guo:** Conceptualization, Data curation, Formal analysis, Investigation, Methodology, Project administration, Validation, Visualization, Writing – original draft, Writing – review & editing. **Mike Lingjue Wang:** Conceptualization, Data curation, Formal analysis, Investigation, Methodology, Validation, Visualization, Writing – review & editing. **Michaela Schwaiger-Haber:** Formal analysis, Methodology, Writing – review & editing. **Xiangfeng Niu:** Investigation. **Shanshan Zhang:** Investigation. **Leah P. Shriver:** Formal analysis, Writing – review & editing. **Gary J. Patti:** Conceptualization, Funding acquisition, Project administration, Resources, Supervision, Writing – review & editing.

## Declaration of competing interest

The authors declare the following competing financial interest(s): The Patti laboratory has a research collaboration agreement with Agilent Technologies. G.J.P. is a scientific advisor for Cambridge Isotope Laboratories and is the Chief Scientific Officer of Panome Bio.

## Data Availability

Data will be made available on request.
