# Supplementary material for: Rapid quantitation of NAD+/NADH and NADPH/NADP+ with mass spectrometry by using calibration constants
Source: Redox Biol. 2025 Nov 29;89:103953. doi: 10.1016/j.redox.2025.103953 (PMC12765170; doi:10.1016/j.redox.2025.103953)
Supplement: Multimedia component 1 [file mmc1.pdf]

Supplementary Information

Rapid quantitation of NAD^+/NADH and NADPH/$NADP^+$ with mass spectrometry by using calibration constants

Qiuyuan Guo[1,2,3], Mike Lingjue Wang[1,2,3], Michaela Schwaiger-Haber[1,2], Xiangfeng Niu[1,2], Shanshan Zhang[1], Leah P Shriver[1,2], Gary J Patti[1,2,4]

1 Departments of Chemistry, Genetics, and Medicine, Washington University, St Louis, MO, USA
2 Center for Mass Spectrometry and Metabolic Tracing, Washington University, St Louis, MO, USA
3 These authors contributed equally
4 To whom correspondence should be addressed: gjpattij@wustl.edu

Supplementary Figures

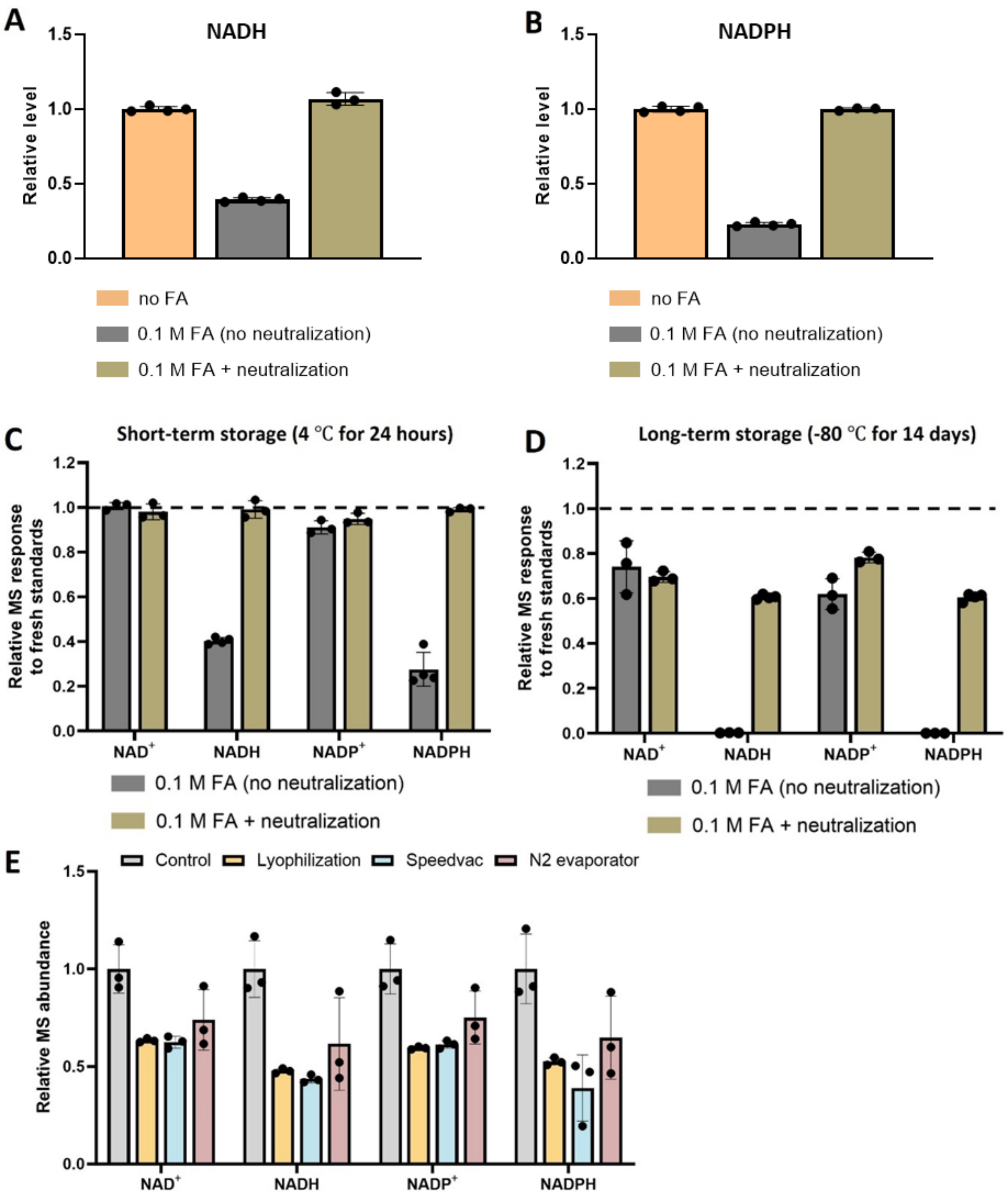

Supplementary Figure 1. Establishing a workflow for LC/MS analysis.
(A-B) Rapid neutralization of acidic extraction conditions prevents the degradation of NADH and NADPH. Although NADH and NADPH are known to be acid labile, the addition of 0.1 M formic acid (FA) during extraction has been shown to quench enzyme activity and prevent the interconversion of oxidized and reduced nucleotides [23]. To test whether neutralization of acidic conditions with ammonium bicarbonate prevents NADH and NADPH degradation in our experiments, we compared standards from each

condition. First, we prepared NADH and NADPH in 2:2:1 of acetonitrile, methanol, and water. We then immediately evaluated the stock solutions by LC/MS. No formic acid was added to this condition, which served as a control. Next, we similarly prepared two sets of NADH and NADPH in 2:2:1 acetonitrile, methanol, and water containing 0.1 M FA. For one of the two sets, we added 2 M ammonium bicarbonate (23:2 v/v) to neutralize the FA and achieve a final pH of 8.5 after 5 min of incubation (matching our extraction workflow). The acid-treated and acid-neutralized samples were then stored at 4 °C for 24 h prior to LC/MS analysis, which matched the handling of samples throughout this study. The preparation of each condition tested here was coordinated so that all of the LC/MS measurements were completed at the same time. In line with prior work [23], the data demonstrate that neutralization effectively limits acid-catalyzed degradation of **(A)** NADH and **(B)** NADPH. **(C)** Stability test of oxidized and reduced cofactors after storage at 4 ℃ for 24 hours or **(D)** after storage at -80 ℃ for 14 days. For **(C-D)**, standards were prepared in 2:2:1 acetonitrile, methanol, and water with 0.1 M FA or in 2:2:1 acetonitrile, methanol, and water with 0.1 M FA that was rapidly neutralized with 2 M ammonium bicarbonate (23:2 v/v to achieve a final pH of 8.5). Neutralization with ammonium bicarbonate occurred immediately after 5 minutes of incubation. The samples, denoted as 0.1 M FA (no neutralization) or 0.1 M FA + neutralization, were then analyzed by LC/MS immediately or stored for either 24 hours or 14 days. For each condition, data were collected for n = 3-4 replicates. The preparation of standards was coordinated so that all of the conditions could be evaluated by LC/MS at the same time. The hashed line represents the average value of the samples evaluated immediately after preparation. The results show that acid neutralization effectively prevents cofactor degradation over 24 hours but not 14 days. **(E)** Stability test of oxidized and reduced cofactors during drying. Samples were dried by using the following instruments: a lyophilizer (Labconco, 700402000), a SpeedVac (Thermo Fischer, SPD1010P1-115), or an N_2 evaporator (Glas-Col, 109A YH-2). The dried standards were then reconstituted in 40 μL neutralized 2:2:1 acetonitrile:methanol:water. All samples were compared to a freshly prepared control that did not undergo drying, n=3. All data are presented as means ± SD.

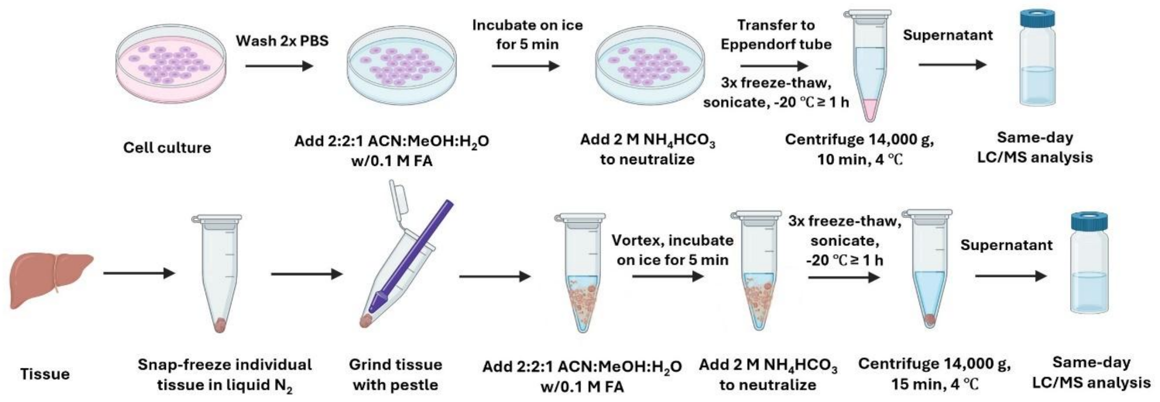

Supplementary Figure 2. Workflow for extracting metabolites from cells in culture or from tissue. Each step is described with text above the arrows.

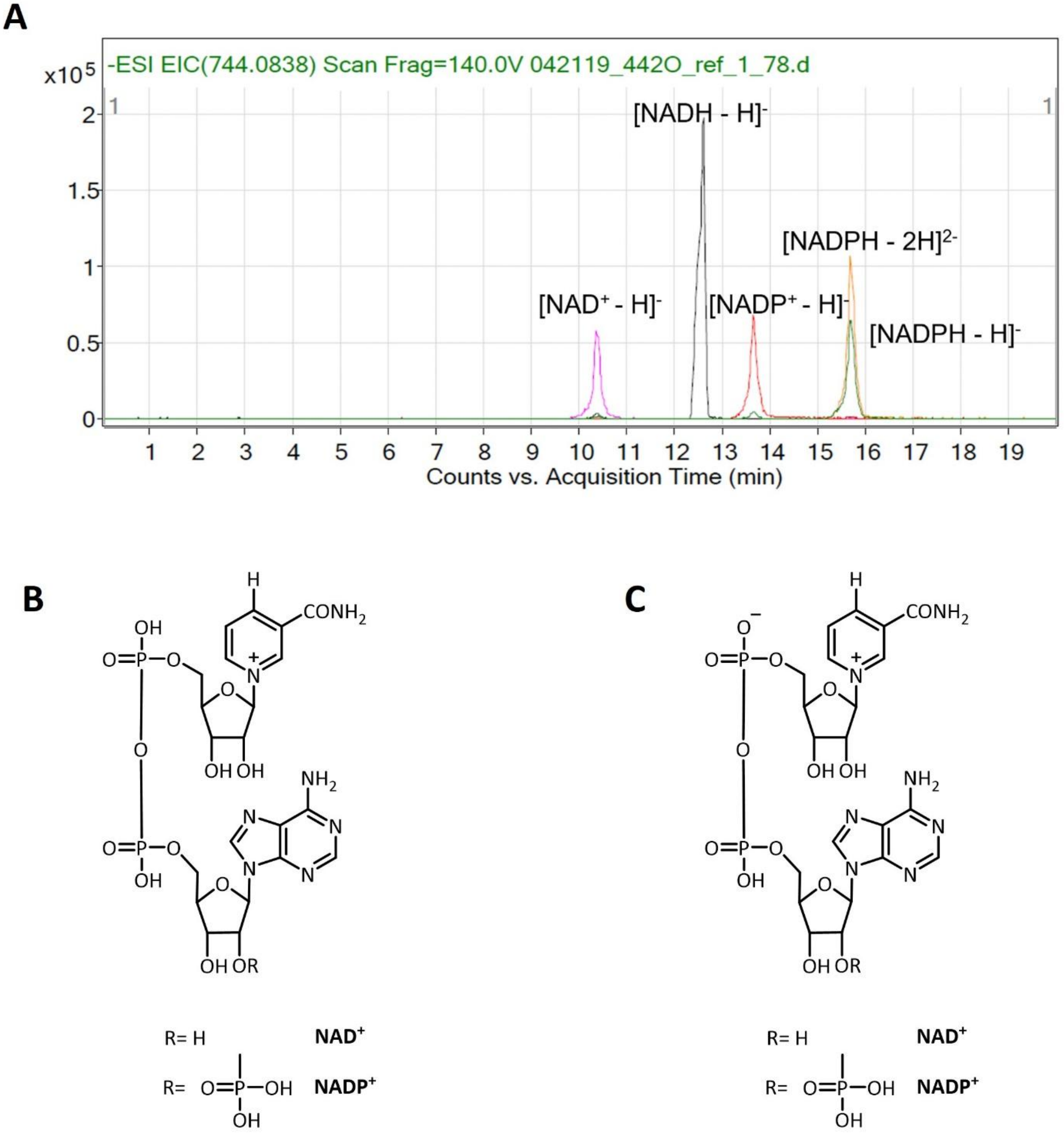

Supplemental Figure 3. Oxidized and reduced cofactors are chromatographically resolved.
(A) Extracted ion chromatograms of each oxidized and reduced cofactor. Separation was accomplished by using hydrophilic interaction liquid chromatography. **(B)** Structure of neutral NAD^+ ($C_{21}H_{27}N_7O_{14}P_2$) and $NADP^+$ ($C_{21}H_{28}N_7O_{17}P_3$), where M denotes the neutral molecule and the detected anion is $[M - H]^-$. **(C)** Structure of positively charged NAD^+ ($C_{21}H_{28}N_7O_{14}P_2$) and $NADP^+$ ($C_{21}H_{29}N_7O_{17}P_3$), where M denotes the +1 charged molecule and the detected anion is $[M - 2H]^-$.

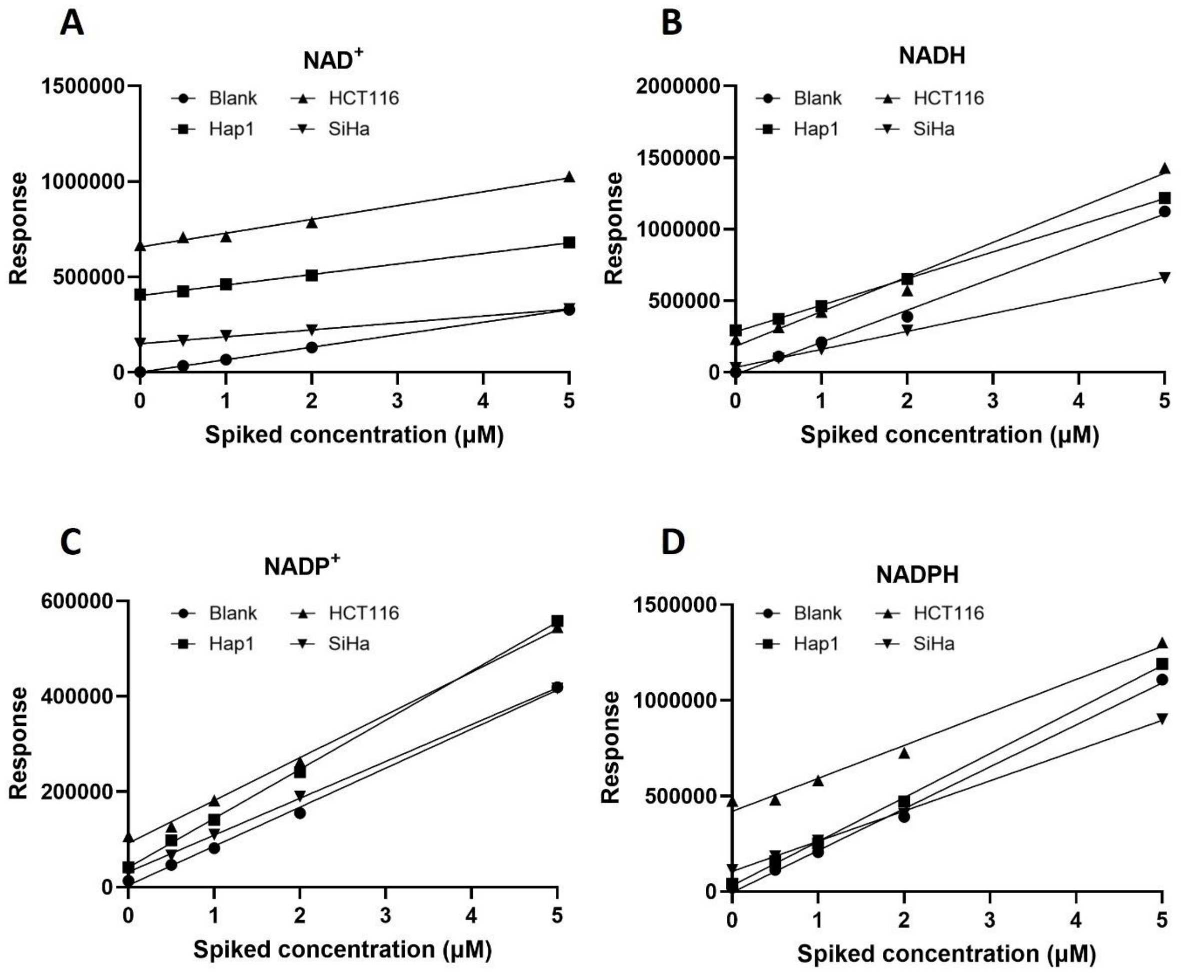

Supplementary Figure 4. Assessing cofactor response across four cell lines.
First, 5 µL of 0, 5, 10, 20, and 50 μM NAD(P) standard mixtures were spiked in 45 µL of metabolite extracts or blanks to achieve a final spiked concentration of 0, 0.5, 1, 2, and 5 μM, respectively. The blanks consisted of 2:2:1 ACN:MeOH:H_2O with 0.1 M formic acid, neutralized by 2 M ammonium bicarbonate (23:2 v/v to achieve a final pH of 8.5). No biological material was present in blanks. Calibration curves are based on standard-addition data from **(A)** NAD^+, **(B)** NADH, **(C)** $NADP^+$, and **(D)** NADPH. Data were acquired on an Agilent 6540 QTOF.

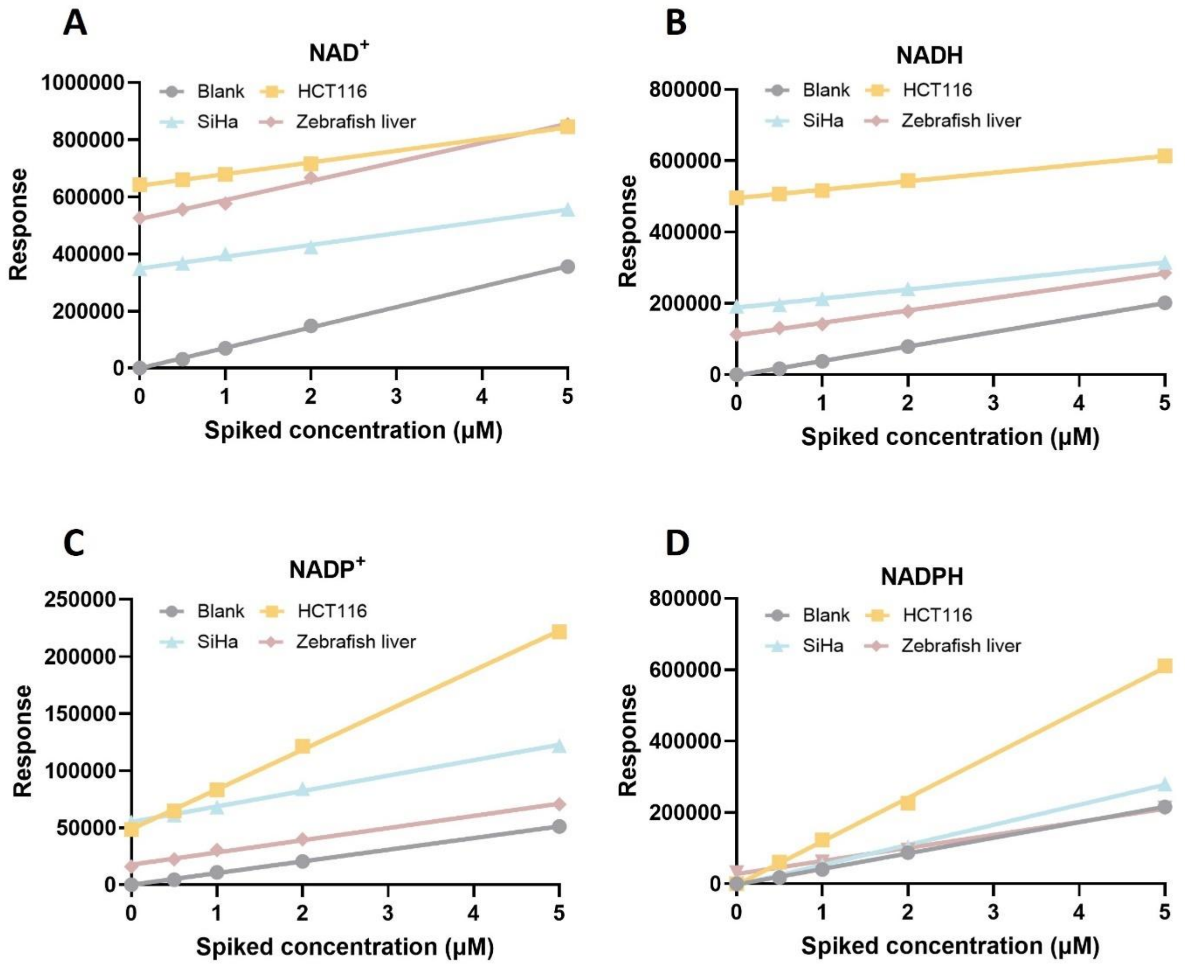

Supplementary Figure 5. Assessing cofactor response across different cells and tissues.
First, 5 μL of 0, 5, 10, 20, and 50 μM NAD(P)(H) standard mixtures were spiked in 45 μL of metabolite extracts or blanks to achieve a final spiked concentration of 0, 0.5, 1, 2, and 5 μM, respectively. The blanks consisted of 2:2:1 ACN:MeOH:H_2O and 0.1 M formic acid, neutralized by 2 M ammonium bicarbonate (23:2 v/v to achieve a final pH of 8.5). No biological material was present in the blanks. Calibration curves are based on standard-addition data from **(A)** NAD^+, **(B)** NADH, **(C)** $NADP^+$, and **(D)** NADPH. Data were acquired on an Agilent 6545 QTOF.

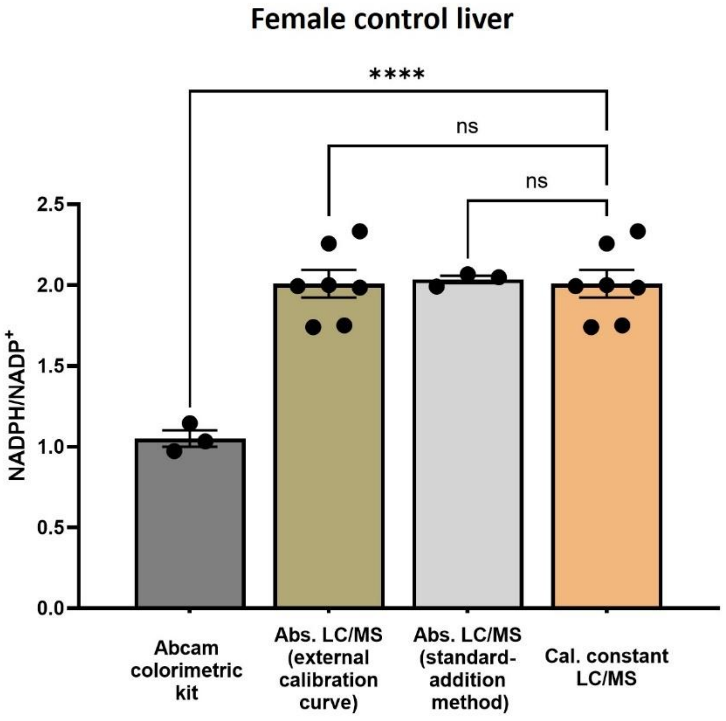

Supplementary Figure 6. Ratios determined by external calibration curves are the same as those determined by the standard-addition method.

NADPH/NADP$^+$ as measured from female zebrafish liver tissue. Each sample was analyzed by using Abcam colorimetric kits or by LC/MS. For LC/MS, redox ratios were determined from absolute concentrations (Abs. LC/MS) or calibration constants (Cal. constant LC/MS). Absolute concentrations were determined either by using external calibration curves or by using the standard-addition method. LC/MS data were acquired from an Agilent 6545 QTOF, n=3-7. Of note, there is no difference between the values measured by external calibration curves and the standard-addition method. All data are presented as means ± SEM. ns = not significant , ****p < 0.0001 by one-way ANOVA.

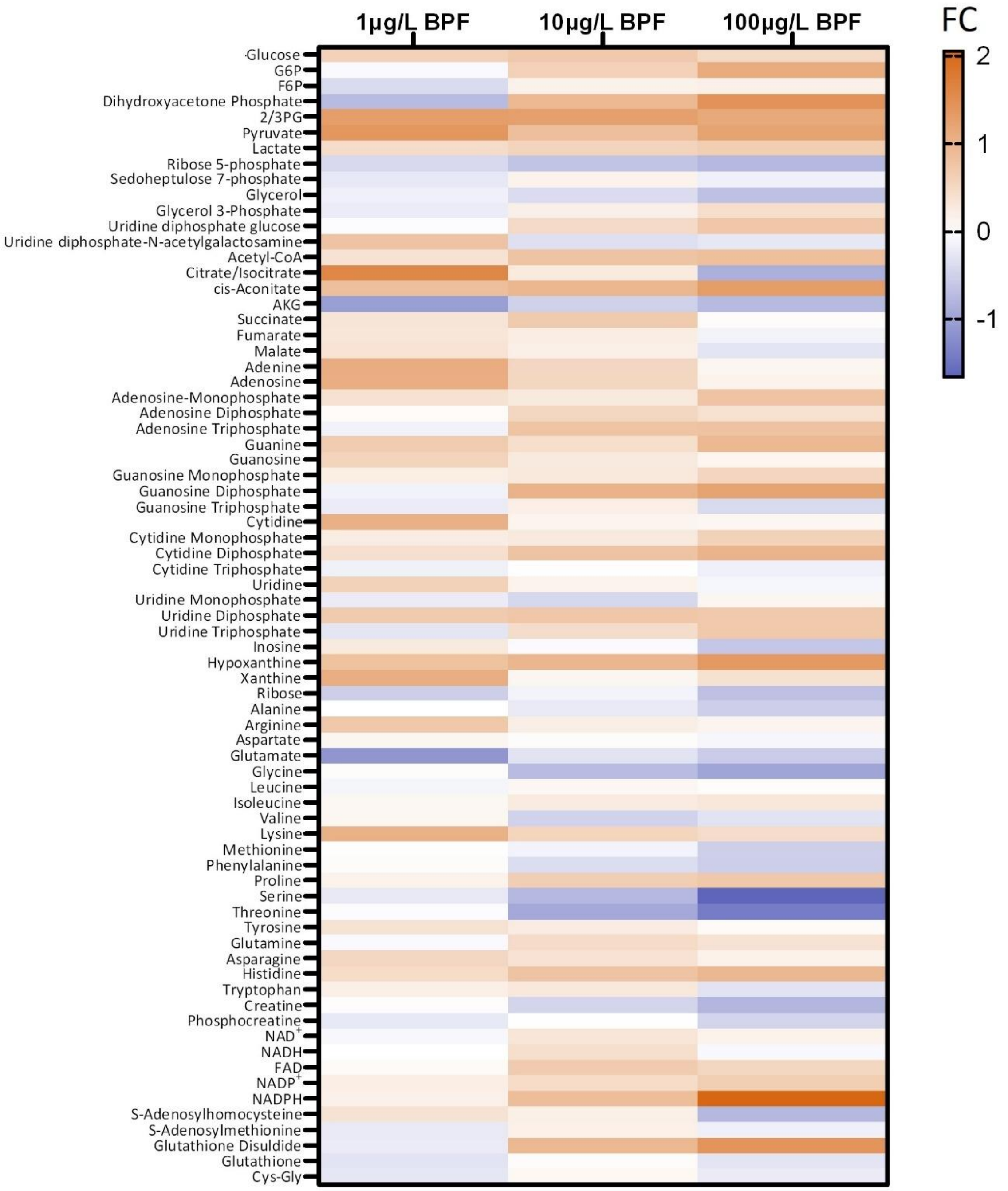

Supplementary Figure 7. Representative metabolomics data that accompanies redox ratios measured by LC/MS.

Heatmap of metabolite levels that change in female fish liver treated with different concentrations of BPF relative to control female liver, n = 7-8.

Supplementary Tables

Supplementary Table 1. LC/MS method characteristics for NAD^+, NADH, $NADP^+$, and NADPH

Compound	Ions and *m/z*	Retention time (minutes)	Linear range (μM)	R^2
NAD^+	$[M - H]^-$ (662.103)	10.22	~ 0.02 to > 50	0.9979
NADH	$[M - H]^-$ (664.118)	12.51	~ 0.02 to > 50	0.9958
$NADP^+$	$[M - H]^-$ (742.068)	13.64	~ 0.02 to > 50	0.9954
NADPH	$[M - H]^-$ (744.084)<br>$[M - 2H]^{2-}$ (371.538)	15.65	~ 0.02 to > 50	0.9969

Supplementary Table 2. Recovery % of $NADP^+$ and NADPH spiked into HCT 116 cell extract as measured by LC/MS and colorimetric assays

Measurement approach	Recovery of NADPH	Recovery of $NADP^+$
Colorimetric assay	118 %	91 %
LC/MS	112 %	94 %

Supplementary Table 3. Calibration constants for various Agilent instruments

Instrument	C_{NAD}	RSD%	R^2	C_{NADP}	RSD%	R^2
6460 QQQ	2.94	33%	0.96	0.42	14%	0.94
6530 QTOF	1.31	7%	0.99	0.37	16%	0.99
6540 QTOF	3.41	7%	0.98	0.46	5%	0.98
6545 QTOF	0.57	4%	0.99	0.26	11%	0.98

Supplementary Table 4. Relative error between true values and the experimentally measured NADPH/$NADP^+$ from standard mixtures and spiked yeast extracts

1:1		10:1		1:10	
Std mixture	Yeast extract	Std mixture	Yeast extract	Std mixture	Yeast extract
-4.30%	-1.63%	-2.35%	-0.91%	0.22%	-1.27%

Supplementary Table 5. Calibration constants from different matrices measured on an Agilent 6540 QTOF

Matrix	k_{NAD+}	k_{NADH}	C_{NAD}	k_{NADP+}	k_{NADPH}	C_{NADP}
Blank	21809	74694	3.42	33254	72957	0.46
Hap1	18415	62051	3.37	31324	76650	0.41
HCT116	24120	80601	3.34	29089	57467	0.51
SiHa	12091	41791	3.46	25748	52616	0.49
Average	/	/	3.40	/	/	0.46
RSD%	27%	27%	2%	11%	18%	9%

Supplementary Table 6. Calibration constants from different matrices measured on an Agilent 6545 QTOF

Matrix	k_{NAD+}	k_{NADH}	C_{NAD}	k_{NADP+}	k_{NADPH}	C_{NADP}
Blank	23838	13544	0.57	3709.5	14544	0.25
HCT116	13606	7876.2	0.58	10587	40606	0.26
SiHa	13740	8431	0.61	4506.6	18747	0.24
Zebrafish liver	22243	11556	0.52	3563.5	12188	0.29
Average	/	/	0.57	/	/	0.26
RSD%	30%	26%	7%	68%	60%	9%

Supplementary Table 7. Relative error between the true value of NADPH/NADP$^+$ and the experimentally measured ratio as determined by using different calibration strategies

1:1			10:1			1:10		
Abs.LC/MS	Cal.constant LC/MS	Peak area ratio	Abs.LC/MS	Cal.constant LC/MS	Peak area ratio	Abs.LC/MS	Cal.constant LC/MS	Peak area ratio
0.46%	2.50%	294.25%	-8.37%	-6.50%	259.62%	0.93%	2.99%	296.11%
